# The effect of hydro-alcoholic extract of *Artemisia absinthium* on appetite in male rats

**Published:** 2015

**Authors:** Sara Baghban Taraghdari, Mohsen Nematy, Mohsen Mazidi, Maryam Kamgar, Mohammad Soukhtanloo, Mahmoud Hosseini, Hassan Rakhshandeh, Abdolreza Norouzy, Habibollah Esmaily

**Affiliations:** 1 *Biochemistry and Nutrition Research Center andDepartment of Nutrition, Mashhad University of Medical Sciences, Mashhad, Iran*^*. *^; 2 *Institute of Genetics & Developmental Biology, Chinese Academy of Sciences, Beijing, China.*; 3 *Department of Physiology, School of Medicine, Mashhad University of Medical Sciences, Mashhad, Iran*; 4 *Pharmacological Research Center of Medicinal Plants, School of Medicine, Mashhad University of Medical Sciences, Mashhad, Iran*; 5 *Health Sciences Research Center, Department of Biostatistics and Epidemiology, School of Health, Mashhad University of Medical Sciences, Mashhad, Iran*

**Keywords:** *Artemisia absinthium*, *Appetite*, *Weight loss*, *Orexigenic effect*

## Abstract

**Objectives:**

weight loss as a consecution of losing appetite in post-operative patients and those suffering from HIV, cancer, cachexia and inflammatory diseases are the main inducements of morbidity and mortality. There is an increasing demand for more efficacious and endurable appetite stimulating treatment for patients with cachexia. Health economics is influenced by the malnutrition which was accounted for 5% of Iranian populations in 2011. *Artemisia absinthium* is known as an orexigenic herb in Iranian traditional medicine. Little evidence is available about its orexigenic effect and mechanism. So, the present study evaluated the possible effect on appetite of hydroalcoholic extract of *Artemisia absinthium.*

**Materials and Methods:**

Thirty male Wistar rats were randomly divided into five groups. Vehicle group received 0.5 ml water per day, control group did not receive anything and other 3 groups received 50, 100 and 150 mg/kg of *Artemisia absinthium* for 7 days respectively. The daily amount of the food eaten by each rat was measured for 10 consecutive days. The amount of energy intake for each rat was also calculated for 7 days during the intervention. The difference in energy intake was calculated and compared between groups.

**Results:**

The results suggest that there was no significant (p>0.05) differences in energy received before and during intervention between three case groups compared with the control group. The energy intake in 1-2 hours after extract injection in all groups, and energy intake after 24 hours interval in third case group (receiving 150 mg/kg extract) is higher compared to other intervals, but it is not significant (p>0.05). So, it can be stated that there was no significant differences between energy intake of 3 case groups and control group.

**Conclusion:**

*Artemisia absinthium* had no positive and dose-related effects on appetite of rats. Future studies are needed to evaluate the orexigenic effect of this plant.

## Introduction

Anorexia is a major risk factor for malnutrition which is caused by various factors (de Groot et al., 2000 ▶). A review proposed that 10% to 40% of hospitalized adult patients demonstrated some degrees of nutritional decline and weigh loss during the hospital stay ( Corish and Kennedy, 2000 ▶ ). Use of traditional medicine to overcome the anorexia, weight loss and malnutrition is safe, effective and economic (Kamboj, 2000 ▶). Most people have experienced a temporary loss of appetite at some time. This is rarely a worrisome symptom unless it lasts for more than a day or two. It can also be a sign of a serious underlying condition, such as depression, cancer or cachexia. It also commonly occurs during a sudden illness, such as an infection (Chapman and Nelson, 1994 ▶). When loss of appetite continues for a long time, a person is at risk for malnutrition and micronutrients deficiencies (Brownie, 2006 ▶; Chandra et al., 1991 ▶). 

Peptide hormones released from the stomach have been described to affect appetite and may play a role in altered food intake in hospitalized patients (Nematy et al., 2005 ▶).

*Artemisia absinthium*, a member of the Compositae, has long been used in diets and in traditional medicine for management of diseases, including diabetes and hepatitis (Cefalu et al., 2008). It possesses anti-diabetic and anti-hyperlipidemic activities in diabetic patients and rats (Noori and DawoodAl-Waili, 1986 ▶; Osawa, 1999 ▶). It has a high content of phytochemicals such as total phenolic composites and total flavonoids, proposing that these compounds contribute to the anti-oxidative activity (Čanadanović-Brunet et al., 2005 ▶). Phenolic materials such as flavonols, cinnamic acids, coumarins and caffeic acids or chlorogenic acids are supposed to have antioxidant properties that might play a significant role in defending cells and any organ from oxidative degeneration (Osawa, 1999 ▶; Wiseman et al., 2000 ▶).

So far, there has been little information about a potential role of *Artemisia absinthium* in the regulation of appetite. To the best of our knowledge, no studies have been published linking energy intake with the *Artemisia absinthium*. For this reason, this paper seeks to address the following questions: can *Artemisia absinthium* elevate food intake in rat? Can *Artemisia absinthium* manipulate the level of energy intake in rat?

## Methods and Materials

**Animals**

Thirty male Wistar rats 8 weeks old and weighing 200-220 g were used. Laboratory temperature was 22±1 °C with 12 hours light/dark cycle, and all rats had *ad libitum *access to food and water. The rats were group housed.

**Foods**

 Laboratory animal food was provided by Javaneh Khorasan Company, Mashhad, Iran. It was constituted of 21% protein and 6.5-7% fat. Every 1000 g of food contained 2750 Kilocalories energy. 

**Preparation of Hydro-alcoholic Extract**

*Artemisia absinthium* was provided by herbarium of Mashhad University. The whole plant was dried at room temperature in shadow, and then powdered. The particle size was not greater than 1/8 inch. Maceration extraction method was used for extraction. The powder was soaked in alcohol 70% as the solvent in a tightly closed container for 2-4 days. It was shaken one or two times a day. The solution was filtered and solute was separated by the use of rotary evaporator. After that, the extract was dried in oven under 40 °C. The dried extract was dissolved in distilled water to prepare relative concentrations before gavage every day.

**Procedure**

Thirty male Wistar rats were randomly selected and divided into five groups (n for each group = 6). Before intervention, 24-hours food intake of each rat for 10 consecutive days was measured while animals had ad libitum access to food and water. To investigate the relationship between *Artemisia absinthium* extract administration and appetite effect, three concentrations of 50, 100 and 150 mg/kg were prepared. These concentrations were administered to the corresponding groups for 7 consecutive days. The rats in the vehicle group received 0.5 ml distilled water every day and those in control group received no intervention. The food consumption in grams by each rat was measured in 1, 2, 4, 6 and 24 hours after extract administration. The average food intake of each rat was converted to Kcal/day. The mean Kcal received in 10 days before intervention, mean Kcal received in 24 hours after extract administration in 7 days of intervention and mean Kcal received after 1, 2, 4 and 6 hours of extract administration were measured. This study was approved by Ethics Committee of Vice Chancellor for Research of Mashhad University of Medical Sciences (MUMS), Mashhad, Iran (A-320, 2010).

**Statistical** **analysis**

 The data was analyzed as Mean±SEM with One-Way ANOVA and Tukey’s test. P-values less than 0.05 were considered to be statistically significant.

## Results

As shown in table 1, there was no significant (p>0.05) differences in energy received before and during intervention between three case groups compared with the control group.

**Table 1:** Mean energy intake in 24 hours before and during intervention in five groups of rats.

**Groups**	**Before intervention (Kcal)**	**During intervention (Kcal)**	**P. value**
First case group (received 50 mg/kg Artemisia solution)	52.7±1.20	52.6±1.86	0.45
Second case group (received 100 mg/kg Artemisia solution)	53.2±1.54	53.0±1.66	0.53
Third case group (received 150 mg/kg Artemisia solution)	53.5±1.23	54.1±1.67	0.63
Control group	53.6±1.32	53.3±1.77	0.53
Vehicle group	53.5±1.23	53.3±1.33	0.21

Values are expressed as a mean±The paired-samples *t*-test is used for comparison between two groups.

**Table 2:** Mean energy intake in distinct time intervals, before intervention in five groups of rats compared with control group.

**Groups**	**Groups**				
	**0-1**	**1-2**	**2-4**	**4-6**	**6-24**
First case group (received 50 mg/kg Artemisia solution)	1.5±0.17	5.5±0.20	4.6±0.62	4.2±0.62	38.7±3.13
Second case group (received 100 mg/kg Artemisia solution)	1.6±0.18	5.5±0.53	4.7±0.91	4.3±0.82	38.3±4.53
Third case group (received 150 mg/kg Artemisia solution)	1.9±0.23	5.8±0.11	5.0±0.65	4.4±0.98	40.2±3.67
Vehicle group	1.4±0.12	5.8±0.16	4.7±0.23	4.8±0.14	37.4±3.23
Control group	1.5±0.11	5.9±0.15	4.5±0.31	5.1±0.17	37.6±3.34

Values expressed as mean ± SEM. The Tukey- Dunnet test was used for comparison with control group.

In table 2 the mean energy intake in time intervals after each extract injection (0-1, 1-2, 2-4, 4-6, 6-24 hrs) in 3 case groups were compared with the control group. Even though the energy intake in 1-2 hours after extract injection in all groups, and energy intake after 24 hours interval in third case group (receiving 150 mg/kg extract) is higher compared to other intervals, but it is not significant (p>0.05). So, it can be stated that there was no significant differences between energy intake of 3 case groups and control group.

## Discussion

Traditional herbal medicines have been used over the centuries before the development of new medicinal molecules and were based on people’s belief and experience. Loss of weight is consequence of poor appetite or anorexia and considered as a major health care issue which can be secondary to various diseases (de Groot et al., 2000 ▶).

Appetite stimulants have been used to overcome decreased appetite. A few examples of stimulants are megestrol acetate (MA), cyproheptadine hydrochloride (CH), cannabinoids, hydrazine sulfate, anabolic hormones, and growth hormone. Currently, the most widely prescribed medication for anorexia is cyproheptadine hydrochloride (Kardinal et al., 1990 ▶). However, the clinical uses of cyproheptadine hydrochloride is limited by its side effects such as somnolence, excitation, hallucinations, ataxia, tachycardia, muscle twitching, occasionally gastric pain, dry mucous surfaces, mydriasis, and rubeosis of the face (Von Mühlendahl and Krienke, 1978 ▶). MA, has substantial side effects and may not be suitable for prolonged use (Homnick et al., 2005 ▶).

Traditionally, *Artemisia absinthium* was used as a sedative, appetite stimulant, analgesic, flavor and anti-parasitic (Lans, 2007 ▶). The committee on herbal medicinal products of European Medicines Agency mentioned the therapeutic indication of *Artemisia absinthium* in temporary loss of appetite with daily dose of 2.3 g of powdered herb in 2-3 doses (EMEA, 2008). This aromatic plant contains up to 0.2% to 1.7% essential oil. Monoterpene thujone is the main constituent of the essential oil (40% - 90%) (Albcn-Puleo, 1978 ▶) which is shown to have anti-convulsion activity by blocking the GABA channels (Höld et al., 2000).

Plants not only provide food and shelter but also serve humanity by preventing and curing different ailments. Herbs and spices have always been useful to cure diseases (Tipu et al., 2006 ▶). The practice of herbal medicine dates back to the very earliest period of known human history. There is evidence of herbs having been used in the treatment of diseases and for revitalizing body system in almost all ancient civilizations, the Egyptian, the Chinese and even Greek and Roman civilizations (Aftab and Sial, 1999 ▶). Currently, there is a high interest in recognition of new potential medications that have minimal side effects which can be used in medicine and the food industry (Srivastava et al., 1993 ▶). 

It was shown that following the oral intake of liquid preparation of *Artemisia absinthium*, gastric secretions, bile and pancreatic enzymes were stimulated and increased dramatically in man (Mills and Bone, 2000 ▶; Bone, 2003 ▶). In the other hand, Batterham et al. (2003) ▶ indicated that pancreatic polypeptide reduces appetite and food intake in humans.

In the present study, as shown in the table 1, there was no significant difference between energy received before and during intervention in 3 case groups compared with the control group. In the table 2, the mean energy intake in time intervals after each extract injection (0-1, 1-2, 2-4, 4-6, 6-24 hrs) in 3 case groups were compared with the control group. Even though the energy intake in 1-2 hours after extract injection in all groups, and energy intake after 24 hours interval in third case group (receiving 150 mg/kg extract) is higher compared to other intervals, but it is not significant (p>0.05). So, it can be stated that there was no significant differences between energy intake of 3 case groups and control group. This could explain that *Artemisia absinthium* had no effect on the appetite of rats. 

Returning to the questions mentioned at the beginning of the article, it is now possible to state that intervention with *Artemisia* solution hydro-alcoholic extract cannot elevate dramatically the level of food and energy intake in rats. Taken together, yet more research on this topic needs to be undertaken to confirm the appetizing mechanism of the plant and possible pathways or hormone interfering with it.
